# A Spotlight on Rad52 in Cyanidiophytina (Rhodophyta): A Relic in Algal Heritage

**DOI:** 10.3390/plants8020046

**Published:** 2019-02-19

**Authors:** Angelo Del Mondo, Manuela Iovinella, Milena Petriccione, Angelina Nunziata, Seth J. Davis, Diana Cioppa, Claudia Ciniglia

**Affiliations:** 1 Department of Biology, University of Naples Federico II, Via Cinthia 21, 80126 Naples, Italy; angelo.delmondo@unina.it (A.D.M.); dianacioppa@gmail.com (D.C.); 2 Department of Biology, University of York, York YO105DD, UK; mi676@york.ac.uk (M.I.); seth.davis@york.ac.uk (S.J.D.); 3 CREA, Council for Agricultural Research and Economics, Research Centre for Olive, Citrus and Tree Fruit (OFA), Via Torrino 2, 81100 Caserta, Italy; milena.petriccione@crea.gov.it (M.P.); angelina.nunziata@crea.gov.it (A.N.); 4 Department of Environmental, Biological and Pharmaceutical Science and Technology, University of Campania “L. Vanvitelli”, 81100 Caserta, Italy

**Keywords:** RAD52, homologous recombination, Cyanidiophytina, *Galdieria*, extremophiles

## Abstract

The RADiation sensitive52 (RAD52) protein catalyzes the pairing between two homologous DNA sequences’ double-strand break repair and meiotic recombination, mediating RAD51 loading onto single-stranded DNA ends, and initiating homologous recombination and catalyzing DNA annealing. This article reports the characterization of RAD52 homologs in the thermo-acidophilic Cyanidiophyceae whose genomes have undergone extensive sequencing. Database mining, phylogenetic inference, prediction of protein structure and evaluation of gene expression were performed in order to determine the functionality of the RAD52 protein in Cyanidiophyceae. Its current function in Cyanidiophytina could be related to stress damage response for thriving in hot and acidic environments as well as to the genetic variability of these algae, in which, conversely to extant Rhodophyta, sexual mating was never observed.

## 1. Introduction

Cyanidiophytina are unicellular red algae living in volcanic and post-volcanic areas, where temperatures rise above 50 °C, and high sulphuric acid concentrations, generated by the oxidation of sulfur gaseous emissions, greatly reduce the pH to values (pH 0.5–3.0) prohibitive for the majority of eukaryotic life forms [1,2,3,4,5,6]. The class includes three genera, the walled *Galdieria* (*G. sulphuraria*, *G. phlegrea*, *G. maxima*) and *Cyanidium* (*C. caldarium*, *C. chilense*) and the naked *Cyanidioschyzon* (*C. merolae*).

The long evolutionary history of Cyanidiophytina began around 1.5 BYA ([7,8,9], before the formation of the supercontinent Rodinia (1.3–0.9 BYA), which resulted in an increase in volcanic activity that would have favored the diversification and dispersal of these thermoacidophilic algae [7,8,9].

According to Gross and Bhattacharya [10], the rising oxygenic atmosphere would have exerted a selective pressure for efficient repair of ROS/UV-damaged DNA, driving ultimately the evolution of sex, through cell-cell fusions, chromosome movement, and the emergence of the nuclear envelope, with the concurrent evolution of meiosis and eukaryogenesis.

The occurrence of meiotic genes is not only related to genetic variation but it is also involved in DNA repair [11]: one of the most threatening forms of DNA damage is the break of the double helix (DSB), as both strands of the DNA duplex are impaired simultaneously. The RAD52 epistasis group is implicated in various cellular processes, such as recombinational repair and chromosome pairing in meiosis, thus guaranteeing the genome integrity; in particular, the RADiation sensitive52 (RAD52) protein catalyzes the pairing between two homologous DNA sequences double-strand break repair and meiotic recombination mediating the loading of RAD51 onto single-stranded DNA ends, and thereby initiating homologous recombination and catalyzing DNA annealing [12] RAD52 is recruited to the Replication Protein A (RPA)-single-stranded DNA nucleoprotein complex, formed upon DSB induction and exonucleolytic ends resection, and mediates its replacement by RAD51. RAD51 then catalyzes strand invasion and D-loop formation. Eventually, RAD52 may assist in capturing the second DNA end and promote its annealing to the D-loop, thus leading to the formation of a Holliday junction [13].

The RAD52 Epistasis Group also includes RAD50, RAD51, RAD54, RAD55, RAD57, RAD59, RDH54, MRE11; they all cooperate in the process of homologous recombination, playing an essential role in the mitotic and meiotic cell cycles, also affecting the response to DNA damaging pro-apoptotic agents [12,14]. Homologs of the RAD52 group of genes have been identified in many eukaryotes, including animals, fungi and plants [15,16,17] and in some cases in prokaryotes [18] indicating high conservation of the recombinational repair pathway. The lack of RAD52 in the vast majority of photosynthetic protists, sexuated or not, is intriguing, considering its role in the homologous recombination process and its relatively high conservation across eukaryotes. Even more unexpected is the presence of this key gene in the asexual red algae *G. sulphuraria* and *C. merolae* genomes along with its absence in other available genomes from sexuated Rhodophyta such as *Porphyra* and *Chondrus*.

The present paper displays the characterization of RAD52 homologs in *Galdieria sulphuraria* genomes. The correspondence of the homologs to yeast and animal of the RAD52 proteins was also provided. In-depth sequence analysis of this protein from 17 *Galdieria* strains was performed in order to delineate its evolutionary relationship and phyletic horizon in available genomes. To exclude a relic nature of RAD52 sequences in *Galdieria*, selective pressures acting on the sequences were detected by analysis of non-synonymous nucleotide substitutions over the number of synonymous substitutions (Ka/Ks) [19,20,21]. The phylogenetic analyses were combined with preliminary gene expression data on *Galdieria* in order to verify increasing RAD52 mRNA expression during saline stress inducing DSBs.

## 2. Results and Discussion

### 2.1. RAD52 Origin and Distribution

**Figure 1:**
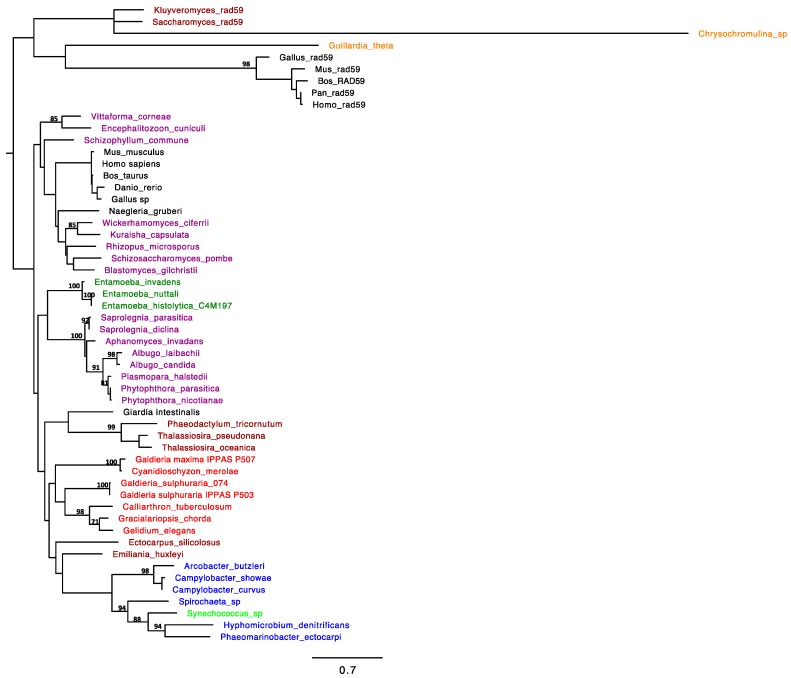
RAD52 homologs, rooted with the RAD52 paralogs outgroup; 140 aligned amino acid sites from 54 taxa were analyzed; this consensus topology derived from >21.000 trees, α = 1.86 (1.45 < α < 2.28), pI = 7.269 × 10^−3^ (7.4239 × 10^−8^ < pI < 0.0217) and lnL = −8952.79. Different colors were used for different taxonomic categories: dark brown, yeasts; orange, cryptophytes; black, mammals; purple, fungi; dark green, amebozoa; light brown, heterokontophytes; blue, bacteria; light green, cyanobacteria.

The RAD52 gene homolog was identified in *G. sulphuraria* 074 genome (Gasu_26690, Accession number M2XIH5). To support the identification of RAD52 homologs within the genome of all analyzed taxa, a phylobayesian inference on protein sequences was built (Figure 1). Analyses showed that all the algal amino acid sequences were strongly supported as homologs of RAD52 excluding then being with RAD59 paralog; by the survey of the sequences, RAD52 appears to be sporadically distributed both among bacteria and eukaryotes. The RAD52 protein is commonly present in Bacteria; among phototrophic bacteria, RAD52 was confirmed only for *Synechococcus sp.* (Cyanophyta) and clusterized with a significant posterior probability (0.99) with *Spirochaete*, *Hyphomicrobium denitrificans*, and *Phaeomarinobacter ectocarpi*. Non-ambiguous blast hits included also Haptophyta (*Emiliania huxleyi*), and Heterokontophyta (*Ectocarpus siliculosus*, *Phaeodactylum tricornutum*, *Thalassiosira oceanica*, *Thalassiosira pseudonana*).

Within the phylogenetic tree, cyanidophycean RAD52 proteins formed a moderately supported clade with the red algal group of Florideophyceae (*Gelidium*, *Gracilariopsis*, and *Calliarthron*), like sister clade of the RAD52 from Heterokonts (*Phaeodactylum tricornutum*, *Thalassiosira oceanica*, *Thalassiosira pseudonana*), with *Ectocarpus* positioned outside of this branch. Noteworthy, all these algal phyla evolved through a secondary endosymbiosis in which a primary red algal cell would have been acquired by a eukaryotic lineage [22]. Previous phylogenetic analyses supported a monophyletic origin of the plastids in cryptophytes, haptophytes, and heterokonts. According to Oliveira and Bhattacharya [23], the plastids of heterokonts would be most closely related to members of Cyanidium-Galdieria group, and not directly related to cryptophytes and haptophyte plastids, thus suggesting for these last an independent origin from different members of Bangiophycidae [23].

According to our investigations, the homology search for RAD52 in green algal genomes gave no results, as well as for land plants, Glaucophyta and Euglenophyta. However, the databases of protein, genomic, and transcribed (EST) sequences from the NCBI queried by Samach et al. (2011) would have provided the evidence of RAD52-like proteins in several plants (monocotids and dicotids), as well as in some ferns and in filamentous (*Spyrogira pratensis*) and multicellular chlorophytes (*Chara vulgaris*). A gene duplication would have occurred according to Samach et al. [24] genome investigations: the green protists S. pratensis and C. vulgaris would possess only the paralog RAD52-1, whilst the gene would be lacking in Stramenopiles, Rhodophytes, and unicellular Chlorophytes.

**Figure 2:**
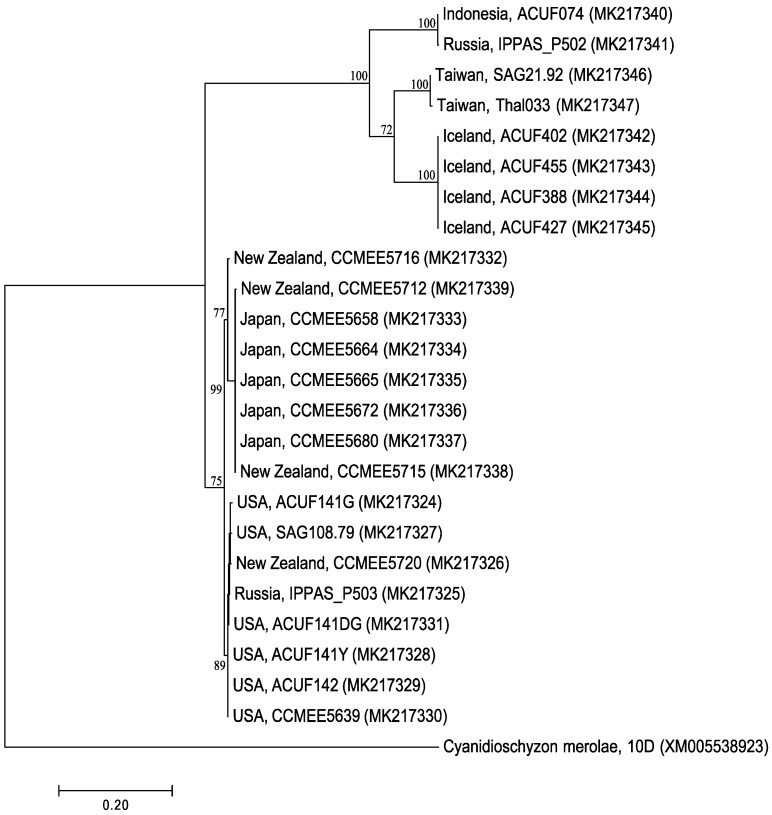
Maximum likelihood tree for 24 newly sequenced *Galdieria* Rad52 genes. Only bootstrap values > 60% were reported.

The level of similarity among RAD52 G. sulphuraria sequences ranged from 72 to 100%; the clustering reflects the phylogeny built on rbcL genes [5]: *G. sulphuraria* from Euroasiatic geothermal sites clusterized in an independent lineage (posterior probability = 0.89), but forming two well supported separate subclades: subclade I, including *G. sulphuraria* from Java and Russia (bp = 100%); subclade II, including both *G. sulphuraria* from Taiwan and *G. sulphuraria* from Iceland (bp = 100%). A second lineage included American accessions of *G. sulphuraria* clustering with Japanese and New Zealand strains, but into two well-supported subclades (Figure 2).

### 2.2. Support for Functional Homology of RAD52 Protein in Cyanidiophytina

**Figure 3:**
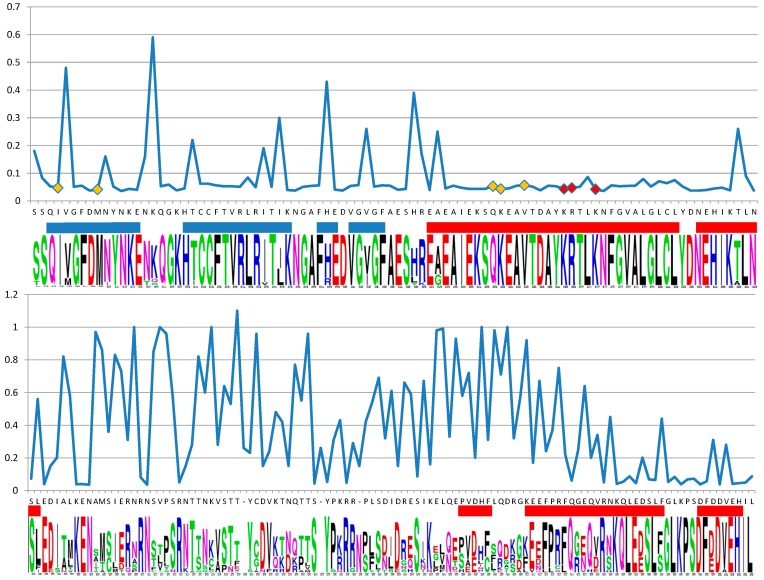
Point value of Ka/Ks ratio along amino acidic sequence indicated by the web-logo graphics. Values gained under the M8 model. Amino acid participating in a β-sheet formation are underlined in blue, while α-helix are underlined in red. All the five AA (I4, M9, Q59, K60, and V63) predicted as DNA binding by I-Tasser are evidenced by a yellow square on the diagram. Residues evidenced by a red square on the diagram are those predicted as DNA binding sites by Kagawa [25] (K129, R130, and R133).

**Figure 4:**
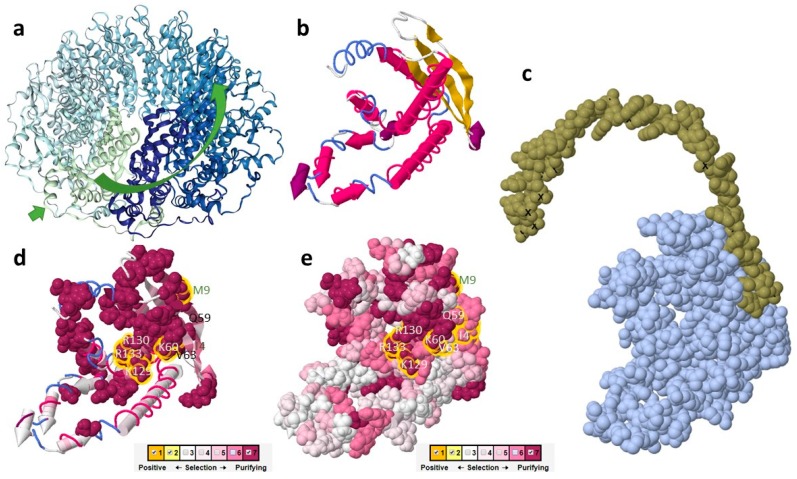
Three-dimensional representation of the structure predicted by I-Tasser integrated with Selecton results; (**a**) structure of human RAD52 is reported with the DNA binding groove evidenced and chains represented in different colors; (**b**) structure predicted by I-Tasser for the reference sequence used in the Selecton analysis; (**c**) DNA binding site as predicted by I-Tasser; (**d**) Selecton results in the M8 model reported on the predicted structure, 3D structures are represented as cartoons with only strongly negatively selected sites highlighted. DNA binding AA is highlighted with yellow halos; (**e**) Selecton results in the M8 model reported on the predicted structure, 3D structures are represented as spacefill. DNA binding AA are highlighted with yellow halos.

The structure of RAD52 from Cyanidiophyceae was modeled on the base of the N-terminal domain of human RAD52 [25]. In Figure 3 and Figure 4 results from selecton analysis are reported and related to information gained by I-Tasser. Results are shown concerning the M8 model. Ka/Ks ratio was never higher than 1, evidencing that no divergent selection was detectable on analyzed fragments. Values by the MEC model were not substantially different (data not shown). The longest conserved sequence was made up of 36 residues that constitute 2 α-helix lining in the inner surface of the DNA binding groove of the protein. Many other highly conserved residues were in the first three β-sheets that constitute the outer surface of the DNA binding groove. In β-sheets, conserved residues were flanked by non-conserved ones. All five AA (I4, M9, Q59, K60, and V63) predicted as DNA binding by I-Tasser had a highly conserved pattern (evidenced by a yellow square in Figure 3 and a yellow halo in Figure 4d,e). For these residues, posterior probability evidenced a confidence interval for Ka/Ks estimated between 2.60 × 10^−5^ and 0.35 for I4 and between 3.20 × 10^−4^ and 0.24 for all the others. Residues evidenced by a red square in Figure 3 and a yellow in Figure 4d,e are those predicted as DNA binding sites by Kagawa [25] (K129, R130, and R133) and were highly conserved as well. The second part of the sequence, not involved in the DNA binding groove formation, seemed not to be under purifying selection during *Galdieria* speciation. In Figure 4c, the predicted model by I-Tasser was shown, based on Singleton et al. [26] partial model for human RAD52 (Figure 4a).

All these features supported the functional homology between RAD52 from Cyanidiophyceae and the known RAD52 protein.

The proteins of RAD52 epistasis group are also involved in the repair of DNA lesions induced by several environmental agents; high salinity represents one of the main injuries inducing DNA damage requiring by the cells the activation of homologous recombination machinery [27]. Salinity, along with drought and desiccation are some of the main stresses challenged by *Galdieria* cells in their hot and acidic environments; our laboratory tests demonstrated the ability of *G. sulphuraria* to tolerate a wide range of NaCl concentrations, from 0.16 to 2.5 M; as a response to salt stress *G. sulphuraria* changed the level of antioxidant enzymes in order to overcome the oxidative stress induced by high salinity (data not shown in the present paper). To evaluate the functionality of RAD52 under salt stress, the gene expression profile of RAD52 of *G. sulphuraria* was analyzed by real-time quantitative polymerase chain reaction (RT-qPCR).

**Figure 5:**
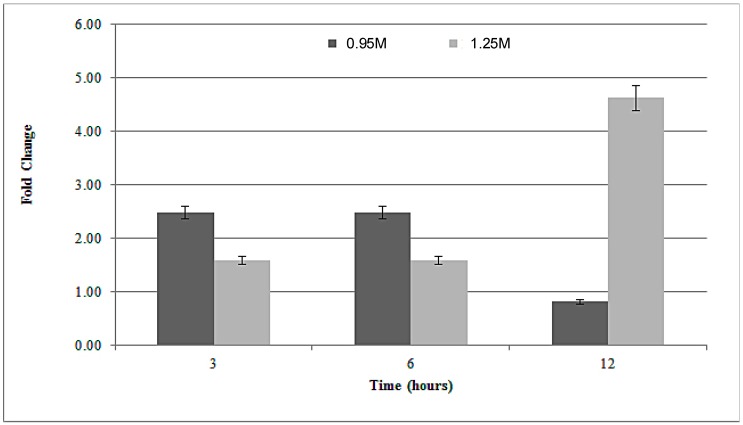
RAD52 gene expression in *G. sulphuraria ACUF 074* cells cultured under 0.95 M (dark grey bars) and 1.25 M (light grey bars) NaCl. The mRNA levels were normalized with respect to the level of mRNA for the reference genes (EF1α and H2B). Bars show means ± standard error (SE) from three independent experiments (n = 3).

RNAs were extracted at multiple points (3, 6 and 12 h) from G. sulphuraria cells under sub-lethal and lethal NaCl (0.95 M and 1.25 M). RAD52 mRNA transcription levels increased after salt-exposition at 1.25 M NaCl with a significant up-regulation at 12 h whereas at 0.95 M NaCl the fold increase was higher compared to the control of up to 6 h’ exposition but then a drastic decrease is observed after 12 h (Figure 5). Accordingly with our expectations, the RAD52 gene is present and plays an important role in *Galdieria*. However, further genetic and biochemical pieces of evidence are necessary on RAD52 functionality and its involvement in DNA repair, along with other proteins of RAD52 groups, generally acting in homologous recombination under stress conditions. The observation of functional conserved residues in a RAD52 protein alignment showed that the catalytic activity of the protein may be conserved not only in *Galdieria* but also in the other related algal organisms.

### 2.3. The Putative Role of RAD52 Protein in Cyanidiophytina

The findings herewith reported show RAD52 homologs in the polyextremophilic red algae Cyanidiophyceae; the conservation of predicted structures and of the amino acid residues implicated in DNA binding strongly supports the hypothesis of a common function between RAD52 from Cyanidiophyceae and the N-terminal domains of RAD52 from previously described proteins. Cyanidiophyceae are likely to be the oldest eukaryote with a RAD52 protein, in which it surely co-operates in DNA damage response and maybe in other meiosis-like mechanisms of genetic variability (not shown); although RAD52 protein is lost for the most part in algae, it looks to be conserved in algal lineages derived from an event of secondary endosymbiosis involving a red alga, in which probably the ancestral RAD52 gene of the internalized rhodophyte was re-arranged and conserved. Because of its key role in DNA repair mechanism, RAD52 could have been retained as a relic heritage in some photosynthetic eukaryotes still living in primordial-like environments, while lost in others, even in closely-related Rhodophyta with intricate life cycles. Being RAD52 gene crucial in meiotic machinery as well, its presence is probably also a hint for looking at sexual behavior in putatively asexual Cyanidiophytina, inhabiting in Archean environments where eukaryogenesis and meiosis co-evolved to reduce the injuries in DNA of a rising oxygen atmosphere.

Interestingly, RAD52 sequences demonstrated to have undergone purifying selection on all the part of the sequence involved in interaction with ssDNA and dsDNA. As expected, mutations in such sites may reduce fitness and are therefore more likely to be removed from the population (purified sites) [28]. In the remaining part of the sequence, instead, several K, R and Y residues are conserved, interspersed in a variable amino acidic context. As evidenced in humans, these parts of the sequence are responsible for the globular structure of each module or RAD52 and of the interactions between modules. In such regions of the protein, a certain sequence variability is compatible with the maintaining of the function.

## 3. Material and Methods

### 3.1. Bioinformatics and Phylogenetic Analysis

RAD52 nucleotide sequences of *G. sulphuraria* 074 (Java, Indonesia) and *Cyanidioschyzon merolae* 10D (Naples, Italy) were retrieved from genome databases [29,30] (http://www.ncbi.nlm.nih.gov/genbank) while 24 additional unannotated nucleotide sequences of RAD52 from different *Galdieria* strains (10 *G. sulphuraria*, 14 *Galdieria* sp.) were obtained by MiSeq Illumina data. RAD52 from *C. merolae* 10D was retrieved from the genome database and used as an outgroup. For DNA extraction used for Illumina, DNA was extracted by resuspending a stationary phase algal paste with DNA extraction buffer [31]. DNA was incubated for 1 h at 65 °C, centrifuged and the supernatant was precipitated by the addition of 1:1 isopropanol. The resultant pellet was suspended in Qiagen buffer, then applied to a miniprep column and washed according to manufacturers’ details. DNA was eluted by adding pre-heated elution buffer provided by Qiagen to the column in 4 sequential elution steps. The sequencing was carried out as reported by Willing et al. [32]. After trimming, Illumina MiSeq reads were assembled using Spades v3.1 [33].

RAD52 amino acid sequences were searched using the National Center for Biotechnology Information (NCBI, http://blast.ncbi.nlm.nih.gov/Blast.cgi) by querying protein, genomic and EST sequences on BLAST. A total of 45 RAD52 protein sequences from different organisms including algae, fungi, animals, and bacteria were recruited and used to generate a multiple sequence alignment, together with 9 RAD59 protein sequences as an outgroup. Among Cyanidiophytina, RAD52 protein sequences were retrieved from genome databases of *G. sulphuraria* 074 (Java, Indonesia), *Cyanidioschyzon merolae* 10D (Japan) (Tables S1 and S2) (http://www.ncbi.nlm.nih.gov/genbank); [29,30] and *G. phlegrea* [34].

Phylogenetic inference of the evolutionary relationships of RAD52 from Cyanidiophyceae and its homologs obtained from public databases was used to verify the orthology of the protein; multiple alignment of amino acid sequences was performed by ClustalW [35], trimmed and adjusted by eye. Only unambiguously aligned amino acid sites were used for phylogenetic analyses. RAD52 phylogeny was rooted by outgroup by using a RAD52 paralogue, RAD59. Bayesian analyses (BA) were performed for combined and individual datasets with MrBayes v.3.1.1 [36] using the Metropolis-coupled Markov chain Monte Carlo (MC3) with the GTR + Γ + I model. For each matrix, one million generations of two independent runs were performed with sampling trees generated every 100 generations. The burn-in period was identified graphically by tracking the likelihoods at each generation to determine whether they reached a plateau.

Maximum likelihood (ML) phylogenetic analysis was performed using the GTR + Γ + I model implemented in RAxML software [37]. Statistical support for each branch was obtained from 1000 bootstrap replications using the same substitution model and RAxML program settings. The RAD52 evolutionary history of *Galdieria* strains was inferred using the maximum likelihood (ML) method, based on the Hasegawa–Kishino–Yano model [38]. A discrete gamma distribution was used to model evolutionary rate differences among sites. Bootstrap analyses were performed as previously described.

### 3.2. In Silico Protein Structure Analysis

The Selecton 2.4 Server (http://selecton.tau.ac.il/) was used to detect selection affecting specific sites. The server program measures the Ka/Ks rate on each amino acid residue [39,40,41]. Both M8 and MEC models were used. In M8 model, each substitution that implies a different coded amino-acid is considered as non synonymous, by contrast, the mechanistic-empirical combination model (MEC) takes into account the differences between amino acid replacement probabilities, expanding a 20 × 20 amino acid replacement rate matrix (such as the commonly used JTT matrix) into a 61 × 61 sense-codon rate matrix. The confidence interval of Ka/Ks values at each site was determined by posterior probability. The I-Tasser server (http://zhanglab.ccmb.med.umich.edu/I-TASSER) was used to predict the 3D structure of the domain and to map DNA binding sites especially conserved on the examined sequences. A multi-alignment representation was draft by using WebLogo application (http://weblogo.berkeley.edu/logo.cgi) and FirstGlance in JMolwas used to visualize the 3D structure (http://bioinformatics.org/firstglance/fgij//index.htm).

### 3.3. Rad52 Gene Expression under Salt Stress

The functionality of the *RAD52* gene was also investigated by analyzing the gene expression profile of the selected meiotic gene under osmotic stress conditions; *G. sulphuraria* ACUF 074 was maintained in liquid culture in Allen medium [42], pH 1.5 at 37 °C under a continuous irradiance of 60 μmol photons m^−2^ s^−1^. When in the exponential growth stage, the culture was supplemented with different NaCl concentrations (0.16–2.5 M). The growth rate was monitored until the stationary phase and evaluated spectrophotometrically at 550nm. All test were prepared in triplicate. Two NaCl stressed *G. sulphuraria* cultures with a sub-lethal (0.95 M) and a lethal (1.25 M) salt concentration were then used to evaluate RAD52 mRNA levels after 3, 6 and 12 hours from the salt addiction. A qRT-PCR assay was performed on *G. sulphuraria* ACUF 074. Total RNA was isolated by PureLink RNA Mini Kit (Thermo Fisher Scientific, Waltham, MA USA), according to the manufacturer’s instructions. The RNA concentration was quantified by measuring the absorbance at 260 nm using a Jasco V-530 ultraviolet-visible (UV/VIS) spectrophotometer (Tokyo, Japan). The purity of all of the RNA samples was assessed at an absorbance ratio of OD260/280 and OD260/230, while its structural integrity was checked by agarose gel electrophoresis. Only high-quality RNA with OD 260/280 and OD 260/230 > 2 was used for subsequent steps. Single-stranded cDNA was synthesized from 100 ng of total RNA using a SuperScript^®^ VILO™ cDNA Synthesis Kit (Thermo Fisher Scientific, Waltham, MA USA), according to the manufacturer’s instructions. EF1α and H2B were used as housekeeping genes [43]. Primer pairs for RAD52 (5′-ACAAGACCTGGACCTTCTCG-3′; 5′-GAAGTCCAACCATCGAAGCC-3′), EF1α (5′-TCGCTCAGGAAAGACAGTTG; 5′-CACAGCAAAACGACCCAAAG-3′) and H2B (5′-GGTACACCCTGACACTGGTA-3′; 5′-CAACTTGCTGGACTCGGAAG-3′) were designed on the *G. sulphuraria* genome. The amplification efficiency of each gene was determined using a pool representing all of the cDNA samples. First, all of the primers were examined by end-point PCR, all of the chosen targets were expressed, and specific amplification was confirmed by a single band of appropriate size in a 2% agarose gel after electrophoresis. In a second step, the pool was used to generate a five-point standard curve based on a 10-fold dilution series. The amplification efficiency (E) and correlation coefficient (R^2^) of the primers were calculated from the slope of the standard curve according to the equation [44]:

$$E(\%)=(10^{\left(-1/slope\right)}-1)\times 100$$

Quantitative real-time PCR was performed using a CFX Connect Real-time PCR Detection System (Bio-Rad, Milan, Italy) to analyze the specific expression of each reference/target gene. cDNA was amplified in 96-well plates using the SsoAdvanced™ SYBR^®^ Green Supermix (Bio-Rad, Milan, Italy), 15 ng of cDNA and 300 nM specific sense and antisense primers in a final volume of 20 µL for each well. Thermal cycling was performed, starting with an initial step at 95 °C for 180 s, followed by 40 cycles of denaturation at 95 °C for 10 s and primer-dependent annealing for 30 s. Each run was completed with a melting curve analysis to confirm the specificity of amplification and lack of primer dimers.

## Supplementary Materials

The following are available online at http://www.mdpi.com/2223-7747/8/2/46/s1, Table S1: Accession numbers of RAD52 amino acid sequences used in this study, Table S2: Accession number of RAD52 nucleotide sequences from Cyanidiophyceae used in this study.
