# Supplementary material for: A Spotlight on Rad52 in Cyanidiophytina (Rhodophyta): A Relic in Algal Heritage
Source: Plants (Basel). 2019 Feb 19;8(2):46. doi: 10.3390/plants8020046 (PMC6410040; doi:10.3390/plants8020046)
Supplement: Supplementary file 1 [file plants-08-00046-s001.pdf]

Table S1. Accession numbers of RAD52 amino acid sequences used in this study.

Taxa	GenBank ID
**RAD52**	
Albugo candida	635369772
Albugo laibachii	325180256
Aphanomyces invadans	673048395
Arcobacter butzleri	315478862
Blastomyces gilchristii	261192601
Bos taurus	528951193
Calliarthron tubercolosum	SRP005182
Campylobacter curvus	516863234
Campylobacter showae	489037738
Candidatus Phaeomarinobacter ectocarpii	918662481
Cyanidioschyzon merolae	544217672
Danio rerio	66269435
Ectocarpus silicolosus	298704860
Emiliania huxleyi	551599108
Encephalitozoon cuniculi	85014303
Entamoeba histolytica	67476176
Entamoeba invadens	471202697
Entamoeba nuttali	672809564
Galdieria sulphuraria IPPAS P507	MK317925
Galdieria sulphuraria IPPAS P503	MK21733250
Galdieria sp. ACUF074	MK217340
Gallus gallus	730466
Gracilaripsis chorda	NBIV01000177
Homo sapiens	863018
Hyphomicrobium denitrificans	505409238
Kuraisha capsulata	584391207
Mus musculus	261824011
Naegleria gruberi	290981385
Phaeodactylum tricornutum	219126773
Phytophthora nicotianae	970651832
Phytophthora parasitica	566015423
Plasmopara halstedii	953492183
Rhizopus microsporus	729702307
Saprolegnia diclina	669164116
Saprolegnia parasitica	813177361
Schizophyllum commune	302678737
Schizosaccharomyces pombe	19112088
Spirochaeta sp.	917473204

Synechococcus sp.	494162898

Taxa	GenBank ID
**RAD52**	
Thalassiosira oceanica	397635710
Thalassiosira pseudonana	220968365
Vittaforma corneae	667640414
Wickerhamomyces ciferrii	754409763
**RAD59**	
Bos taurus	61864423
Chrysochromulina sp.	922864786
Gallus gallus	45383087
Guillardia theta	551643257
Homo sapiens	21717826
Kluyveromyces lactis	49643317
Mus musculus	13385116
Pan troglodytes	55645233
Saccharomyces cerevisiae	6320144

Table S2. Accession number of RAD52 nucleotide sequences from Cyanidiophyceae used in this study.

Strain	Strain code	Accession number
Galdieria sulphuraria	ACUF141G	MK217324
	ACUF141Y	MK217328
	ACUF141DG	MK217331
	ACUF142	MK217329
	ACUF388	MK217344
	ACUF402	MK217342
	ACUF427	MK217345
	ACUF455	MK217343
	SAG108.79	MK217327
	SAG21.92	MK217346
Galdieria sp.	IPPAS_P503	MK217325
	CCMEE5720	MK217326
	CCMEE5639	MK217330
	CCMEE5716	MK217332
	CCMEE5658	MK217333
	CCMEE5664	MK217334
	CCMEE5665	MK217335
	CCMEE5672	MK217336
	CCMEE5680	MK217337
	CCMEE5715	MK217338
	CCMEE5712	MK217339
	ACUF074	MK217340
	IPPAS_P502	MK217341
	THAL033	MK217347
Cyanidioschyzon merolae	10D	XM_005538923
